# XVII International AIDS Conference: From Evidence to Action - Epidemiology

**DOI:** 10.1186/1758-2652-12-S1-S2

**Published:** 2009-10-06

**Authors:** Martin Flynn, Rodney Kort

**Affiliations:** 1 International AIDS Society, Geneva, 1216 Cointrin, Switzerland; 2 Kort Consulting, Toronto, M4Y 2T6, Canada

## Abstract

As the epidemic matures, accurate information about where new infections are occurring, and in which populations, is becoming increasingly critical in designing effective, targeted interventions relevant to current epidemiological trends. Although the quality and accuracy of HIV surveillance data and methodology have improved, in many cases the second generation WHO/UNAIDS surveillance system has not been fully implemented at the national level. National surveillance systems in many low and middle-income countries often do not collect disaggregated data on some most at risk populations, which is critical to developing targeted prevention interventions.

While the majority of new infections occur in low- and middle-income countries, the dynamic situation in high-income countries demands renewed attention.

## Discussion

### Establishing better epidemiological information

The implications of the epidemiological data presented at AIDS 2008 suggest, as one speaker noted, the need for a substantial recommitment to effectively targeted prevention interventions to address prevention fatigue and to diversify access to HIV testing and counselling [1]. Peter Piot, attending his final International AIDS Conference as UNAIDS Executive Director, warned in the Opening Session: "The epidemic is evolving. HIV infections are rising in some countries where we thought prevention had been successful, and new epidemics are appearing... Let us not forget that the epidemic could still bring us new surprises - as it has done so many times already" [2].

Data from the UNAIDS 2008 Report on the Global AIDS Epidemic, released immediately prior to the conference, indicates that the percentage of people living with HIV globally has remained stable since 2000 (at an estimated 0.8%) and that new infections have declined from 3 million/annum in 2002 to 2.7 million/annum in 2007 [3]. However, overall prevalence, due to ongoing infections and reduced mortality as a result of antiretroviral therapy (ART) rollout, remains high. Thirty-three million people were estimated to be living with HIV at the end of 2007, up from 29.5 million in 2001, and over 7,400 people continue be infected daily, with 2 million AIDS deaths in 2007 alone [4]. Most sub-Saharan African countries are reporting reductions in new infections, although this is partially offset by increases in other regions, particularly among injecting drug users (IDUs), gay and other men who have sex with men (MSM), and sex worker populations.

**Figure 1:**
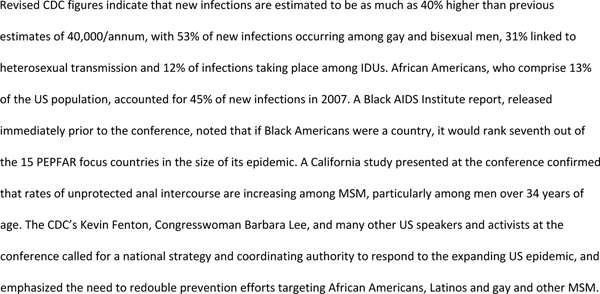
**The US Epidemic**.

The challenges of establishing precise HIV surveillance data to help inform the response and assess the impact of prevention interventions, even among high-income countries, was highlighted by revised estimates published by the United States Centers for Disease Control and Prevention (CDC) shortly before the conference (Figure 1). The US data revealed much higher rates of infection in the US than previously published, thanks to a new technology that is able to detect recent seroconversions. The new figures - and the impact of the epidemic on already marginalized communities in the US - were the subject of a report issued by the Black AIDS Institute issued immediately prior to the conference and resulted in heated debate and activism at the conference itself (see sidebar on the US epidemic) [5]. Other high-income countries, most notably the United Kingdom and Germany, also have seen recent increases in HIV infections over the last few years, concentrated primarily among gay men.

As the epidemic matures, information about where new infections are occurring, and in which populations, is becoming increasingly critical in designing interventions relevant to current epidemiological trends. Although tools to measure HIV incidence would provide the most useful data for both targeting and evaluating prevention interventions, such tools often are unavailable outside of research settings [6]. In his plenary overview of the current spread of HIV, Geoff Garnett further noted that declines in prevalence may, in part, be related to the natural course of an epidemic, and not exclusively the result of widespread risk reduction behaviours [7]. He proposed a conceptual framework for understanding risk that overlays proximate determinants of risk (such as the number of sexual partners and biological factors) with social epidemiology (including social, structural and individual factors) to develop an accurate model of individual and population level risks for HIV infection.

In the same session on current epidemiology, Elizabeth Fadul (Youth Programme Working Group, Dominican Republic) noted that the epidemic is increasingly affecting young people (15 - 25 years of age), who represent 45% of new infections, and high rates of HIV infection among marginalized populations such as MSM, IDUs, sex workers and migrant populations [8]. Additional regional epidemiological data is included in the introduction to each region in Section 6: Regional Focus.

### Inadequate focus on vulnerable populations

Women and girls continue to be disproportionately affected in sub-Saharan Africa, where they represent 60% of PLHIV. Although the ratio of males to females living with HIV globally has remained stable at 50:50 since 2001, women's share of new infections is increasing in several countries [9].

More encouragingly, prevalence among young, pregnant African women (15 - 24 years of age) has dropped significantly, with seven countries meeting or exceeding the 2010 target of a 25% seroprevalence reduction in this key demographic, a target set by the international community in the 2001 Declaration of Commitment on HIV/AIDS.

**Figure 2:**
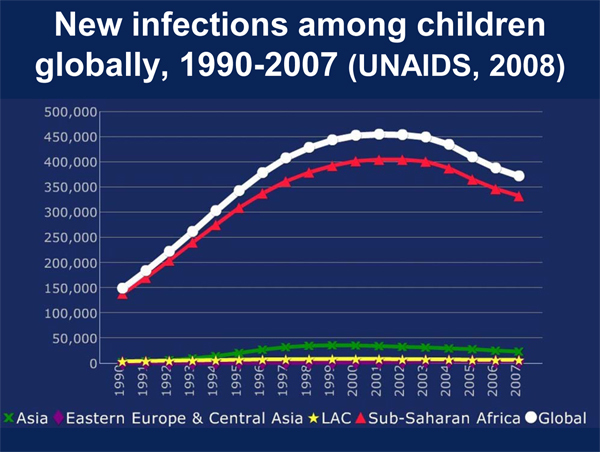
**New infections among children globally, 1990-2007 (UNAIDS, 2008)**. *Source: Richter, L. No Small Issue: Children and Families (WEPL0102)*.

The scale-up of prevention of mother-to-child transmission (PMTCT) using antiretrovirals has increased significantly in recent years, from less than 10% of pregnant women living with HIV covered in 2005 to 34% in 2007, and new infections are declining. However, delegates were reminded that, compared to adults, children remain disadvantaged in terms of treatment access are more vulnerable to the social and familial impacts of AIDS [10,11]. The impact of HIV on children received unprecedented attention at AIDS 2008, most powerfully in the plenary presentation by Linda Richter (Human Science Research Council, South Africa) who argued forcefully that - despite recent gains in attention and resources - children remain underserved and vulnerable to the cascading effects of HIV and AIDS-related mortality on parents, families and communities. While the impact of expanded access to PMTCT interventions is reflected in recent declines in new infections among children (Figure 2), early infant testing is still only available to less than 8% of newborns in low-income countries [12].

### Data quality: implications for most at risk populations

**Figure 3:**
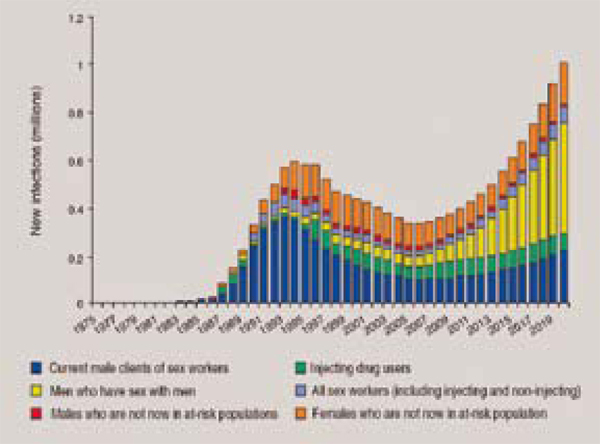
**Early Success, potential future failure and a growing MSM epidemic in Asia**. *Source: Wilson, D. Overview of MSM Epidemiology in the Global South, from *Redefining AIDS in Asia: Crafting an Effective Response, *Commission on AIDS in Asia.*

The disproportionate - and often underreported - impact of the epidemic on gay men and other MSM was the focus of a pre-conference event and was a dominant topic of discussion within both the formal programme and other conference events. At the pre-conference, David Wilson's (The World Bank) state of the art overview of MSM epidemiology in the global South - which also included an analysis of seroprevalence among female sex workers and IDUs - drew attention to the gap between the high prevalence and increasing HIV incidence among many gay/MSM populations and the availability of resources dedicated to this population [13]. This issue is particularly relevant for Latin America, where an estimated 20% of MSM are seropositive, a figure that rises to 30% in the Caribbean. Studies in Africa place seroprevalence among MSM between 20% and 40% although it is difficult to assess the accuracy of these estimates as MSM are often not included in national surveillance systems [14]. Projections of the expanding epidemic among MSM in Asia underscores the potentially disastrous consequences of not delivering effective prevention interventions to this key population (Figure 3).

**Figure 4:**
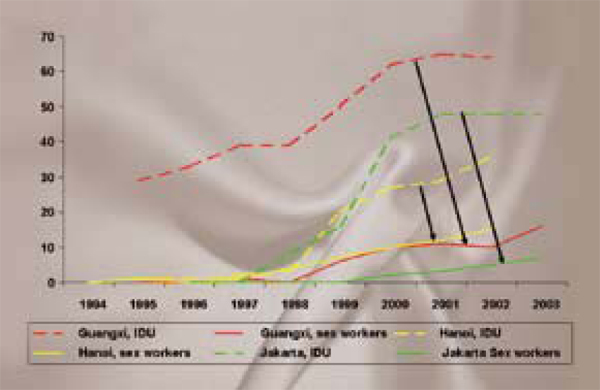
**IDU igniting HIV infection among sex workers in Asia**. *Source: Pisani, E. HIV Infections among IDUs and Sex Workers in Asia*.

Wilson's analysis of population-based seroprevalence studies in several Asian cities also reveals the extent to which rising incidence among IDUs correlates with subsequent increases in HIV prevalence among female sex workers and other populations (Figure 4). If this hypothesis is validated in other cities, it could have enormous consequences for Eastern Europe and Central Asia, where the vast majority of infections are the result of unsafe injecting practices driven by structural factors such as the lack of substitution therapy, a punitive approach to drug use, poor access to drug treatment, and limited access to evidence-based prevention interventions.

## Conclusion

The major issues identified in epidemiological presentations and discussion at the conference focused on three issues, the first two closely related:

First, while the quality and accuracy of HIV surveillance data and methodology have improved, in many cases the second generation WHO/UNAIDS surveillance system has not been fully implemented at the national level. Many countries are still failing to produce precise surveillance data, particularly with respect to highly marginalized populations such as MSM, IDUs and sex workers. While significant work on building core HIV surveillance capacity is progressing, it is clear that the UNAIDS mantra, "know your epidemic", remains a formidable challenge. A recent survey of 153 low- and middle-income countries revealed that only 56 had fully-functioning surveillance systems and 49 had poor performing systems [15].

Second, existing national surveillance systems in many low- and middle-income countries often do not collect disaggregated data on some most at risk populations. If disaggregated data are not collected as part of a comprehensive national surveillance system, there are only a limited number of population-specific studies on which to base estimates of infection and progress in HIV prevention, a point underscored by Kieran Daly (International Council of AIDS Service Organizations, Canada) at a session on tracking progress on UNGASS targets [16]. It is clear that the structural inequalities faced by gay and other MSM, sex workers and IDUs, which have driven much of this epidemic among these populations since it first emerged, continue to hamper national responses to AIDS. In his plenary presentation on MSM, Jorge Saavedra, from Mexico's Centro Nacional para la Prevencion y Control del VIH/SIDA (CENSIDA), noted: "we have failed to bring down the incidence among MSM because, with some exceptions, we have not tried" [17]. The issues he raised about how homophobia continues to undermine the AIDS response was echoed in equally strong statements at the Opening Session by leaders as diverse as Mexican President Felipe Calderon and UN Secretary General Ban Ki-moon. Perhaps additional advocacy efforts targeting political leaders and government officials are required to turn the language former Botswana President Festus Mogae used in his Opening Session speech regarding "people who engage in unusual sexual practices" into an explicit acknowledgment that MSM in Africa - and other regions - have not served them well to date.

Third, while the majority of PLHIV live in low- and middle-income countries and these areas are also home to the vast majority of new infections, the dynamic situation in high-income countries demands continued attention. The new US figures, together with data from other high-income countries with established epidemics, such as Germany and the UK, reveal rising HIV infection rates among gay and other MSM, as well as recent increases in rates of hepatitis C virus (HCV) co-infection. In the Russian Federation and other countries in Eastern Europe and Central Asia, unsafe injecting practices are responsible for the vast majority of new infections, with harm reduction interventions for IDUs facing a daunting legal and policy context.

## Competing interests

Martin Flynn was employed by the IAS during the preparation of this report. Rodney Kort is an independent consultant contracted by the International AIDS Society for the purpose of preparing and editing The AIDS 2008 Impact Report for publication.

## Authors' contributions

Martin Flynn and Rodney Kort drafted the text for this section of the supplement. This manuscript has been approved for publication.
